# Cannabis use and patterns among middle and older aged Canadians prior to legalization: a sex-specific analysis of the Canadian Tobacco, Alcohol and Drugs Survey

**DOI:** 10.1186/s12889-020-10074-z

**Published:** 2021-01-06

**Authors:** Asvini Keethakumar, Vrati M. Mehra, Nazilla Khanlou, Hala Tamim

**Affiliations:** 1 School of Kinesiology and Health Sciences, Faculty of Health, York University, Toronto, Canada; 2 School of Nursing, Faculty of Health, York University, Toronto, Canada

**Keywords:** Cannabis, Marijuana, Substance use, Gerontology, Older adult, Canadian, Legalization

## Abstract

**Background:**

The recreational use of cannabis was legalized across Canada in October 2018. While many people use cannabis without harm, adverse outcomes have been noted in a few populations, including middle-aged and older adults. Given that the current literature has neglected to study cannabis use among this population and between sexes, the objective of our study was to identify the prevalence, characteristics, and patterns of cannabis use among middle and older aged males and females prior to legalization in Canada.

**Methods:**

Secondary analysis was conducted on the Canadian Tobacco, Alcohol and Drugs Survey 2017, with the sample restricted to adults ages 40 and above. The main outcome was defined as past-year cannabis use and statistical analysis was conducted separately for males and females. Bivariate and multivariable logistic regression was performed to identify associations between the main outcome and various sociodemographic, health, and substance use variables. Explanatory supplementary variables were also explored.

**Results:**

In 2017, 5.9% of females and 9.0% of males over the age of 40 reported past-year cannabis use. Almost 62% of males who used cannabis in the past-year reported a failed attempt at reducing or stopping their cannabis use. Over half (56%) of older females, self-reported using cannabis for medical purposes. Additionally, over one in five older adults reported using a vaporizer or e-cigarette as a delivery method for cannabis. Significant characteristics of male cannabis use included having no marital partner, cigarette smoking, and illegal drug use. Furthermore, significant predictors of past-year cannabis use in females included residing in an urban community, Eastern- Atlantic provinces or British Columbia, having fair/poor mental health, smoking cigarettes, use of other tobacco products, and illegal drugs.

**Conclusion:**

To our such knowledge, this is the first nationally representative study to outline the prevalence, characteristics, and patterns of past-year cannabis use prior to Canadian legalization, among middle and older aged Canadians. Results from this study are expected to be used to reliably to track changes in usage, behaviours, and related disorders in the years to come.

## Introduction

As of October 2018, Canada became the second country in the world to legalize the recreational use of cannabis, also commonly known as marijuana, weed, and hashish [1]. According to the Government of Canada, the legalization of cannabis was implemented with three goals in mind; keeping it out of the hands of minors, reducing black market profits, and protecting the health of Canadians by providing access to safe cannabis via secure sources [2]. Prior to legalization, cannabis was the most commonly used illicit drug in Canada [3]. In 2014 alone, cannabis related costs burdened the Canadian economy with upwards of 2.8 billion dollars to combat economic, health and social consequences [3, 4]. While many people use cannabis without harm, it is important to note that adverse outcomes have been reported, especially among aging adults [5, 6]. In some adults, use of cannabis has been linked to an increased risk of psychiatric disorders such as schizophrenia and psychosis [7, 8], and chronic conditions such as heart disease, asthma, pneumonia, cognitive decline, and loss of executive functioning [9–12]. A recent meta-analysis and systematic review noted that close to 1 in 8 people who have used cannabis experience cannabis dependence and cannabis abuse, while over 1 in 5 users may go on to develop a cannabis use disorder [13]. Although cannabis is commonly associated with age-related declines, legalization has been readily accepted by the Canadian public [14, 15].

In 2012, 42.5% of the entire Canadian population reported having ever used cannabis, with 12.2% reporting use in the past year [16]. The prevalence was highest among 18–24-year olds, with 33.3% reporting past-year use, followed by 20% among ages 15–17, 15.6% in ages 25–44, 6.7% in ages 45–64, and 0.8% in those aged 65+ [16]. While cannabis is clearly popular among younger Canadians, major substance use surveys have shown that cannabis use has steadily grown in adults [4, 17]. Studies from the United Kingdom and United States have also shown a similar trend with cannabis being one of the most widely used drugs in adults over the age of 50 [18, 19].

Additionally, cannabis use behaviours among males and females have been noted as distinct [20, 21]. Between 2002 and 2010, Canadian males report a 2.4% increase in their lifetime cannabis use, while use among females remained stagnant [16]. However, more recently, rates of female cannabis use has rapidly approached the rate of males [4]. Because substance use has historically been considered a male dominant problem, much of the literature has neglected to evaluate sex specific differences [22]. Pederson and colleagues (2015) noted that gender inequity among substance use research and health promotion efforts is a global challenge which must be addressed moving forward [23]. As such, it is essential that studies examine the sex specific differences of cannabis use and any related characteristics in Canada and across the globe.

In Canada, sociodemographic and economic factors associated with increased cannabis use include being a male, residing in the provinces of British Columbia and Nova Scotia, dwelling in urban areas, having a lower socioeconomic status, lower levels of post-secondary education attainment, and lower employment rates [6, 16, 24, 25]. Additionally, mental health complications such as depression, emotional distress, and psychosis have been associated with cannabis use [6, 26]. Finally, cannabis use may be linked to the uptake of other substances, such as tobacco, alcohol, and illicit substances such as opioids, amphetamines, and cocaine [6, 26]. Historically, given that younger Canadians have been the highest users of cannabis, most studies have focused on the youth and young adult population [26–28]. These results may not be generalizable to older age groups or specific sexes, as they have been noted to be distinct in the ways they access, use and respond to cannabis [29].

Currently, middle and older aged adults represent more than 51% of the Canadian population, making up a significantly large portion of the demographic [30]. With the recent cannabis legalization and increased access to cannabis products across the nation, the relationship between cannabis use among older Canadians may undergo important changes. In order for the Canadian public health system to evaluate and update policies around legalization in the upcoming years, it is vital that information around cannabis utilization is available and can be reliably tracked. Therefore, the objective of this study is to identify the prevalence, characteristics, and patterns of past-year cannabis use among older Canadians, separately for males and females, prior to cannabis legalization in Canada.

## Methods

### Data source and study population

This study utilized the 2017 Canadian Tobacco, Alcohol and Drugs Survey (CTADS), which was conducted among all Canadian provinces. The CTADS is a cross-sectional survey which collected detailed information on drug use, demographics, and lifestyle factors among Canadians. The survey was conducted biannually by Statistics Canada, in collaboration with Health Canada. The target population of the CTADS consisted of individuals aged 15 years and older, living in all ten provinces of Canada. However, residents of the three Canadian territories, residents of institutions, and those without either a home or cellular phone were excluded from the survey. Data collection started on February 1st, 2017 and concluded on December 31st, 2017. All responses to the survey were voluntary and self-reported directly by each participant. Further details about the survey data collection methods can be found on the Statistics Canada website [31]. For the purpose of this study, the survey responses were limited to include all individuals who were aged 40 and above at the time of response. Where possible, this investigation adhered to STROBE reporting guidelines for observational studies [32].

### Measures

The main outcome for the study was “past-year cannabis use”. This was measured by the question “During the past 12 months have you used marijuana?” The respondents were given the choice of answering “Yes” or “No”.

A wide range of covariates were considered to be potential predictors of cannabis use, categorized by the following: sociodemographic factors, health factors, and substance use variables. These groupings were further categorized to include the following variables: Sociodemographic factors including age, sex, province of residence, type of community dwelling (rural or urban), marital status (partner or no partner), indigenous identity (yes or no), level of education (university degree and above, trade/college, secondary or less than secondary), and current employment (employed or not employed). Health factors comprised of self-perceived general health (excellent/very good, good, fair/poor) and self-perceived mental health (excellent/very good, good, fair/poor). Substance use variables **(**all of which were dichotomized into yes or no) included past 12 month alcohol use, current cigarette smoking, use of tobacco alternatives (containing one or more of cigarillo, cigar, tobacco water pipe or smoke-less tobacco use in the past 30 days), and other illicit drug use (including past 12-month use of one or more substances including cocaine, speed/meth, ecstasy, hallucinogens, salvia, heroin, inhalants, abuse of pain relievers, stimulants and sedatives).

Explanatory supplementary variables were considered, which included self-reported cannabis use for medical purposes (yes or no), main self-reported medical condition cannabis is used for, method of consumption, source of cannabis, frequency of past three-month use, failed attempt at controlling/reducing intake over past 3 months, as well as mean age at first cannabis use.

### Statistical analysis

To achieve the targets of this study, statistical analysis was conducted separately for males and females. Descriptive statistics were conducted on all main and supplementary variables. The relationship between all characteristics and the main outcome were conducted using chi squared tests and binary logistic regressions. Multivariable logistic regression was conducted to adjust for all the covariates. Both unadjusted and adjusted Odds Ratios (ORs) along with the 95% Confidence Intervals (CIs) have been reported. Approximately 4.31% of the cases had missing information and were subsequently excluded from the final analysis. Population weights were applied to each calculated estimate to adjust for sampling methodology and to report unbiased population parameters. Bootstrapping was performed to account for the complex sampling design. Statistical analyses were conducted using Statistical Package for Social Science (SPSS, version 26.0) and STATA version 13, (StataCorp, College Station, TX). Statistical significance for all analyses was set at alpha < 0.05 for a two tailed test.

## Results

Overall a total sample of 4789 individuals were included in this analysis, weighted to represent 8,890,444 females and 8,181,855 males aged 40 and above in Canada. After population weights were added, 60.4% of the respondents were aged 40 and above at the time of the survey. The total prevalence of cannabis use among females was 5.9% as compared to 9.0% in males, for a total of 7.4% in the entire sample.

**Fig. 1:**
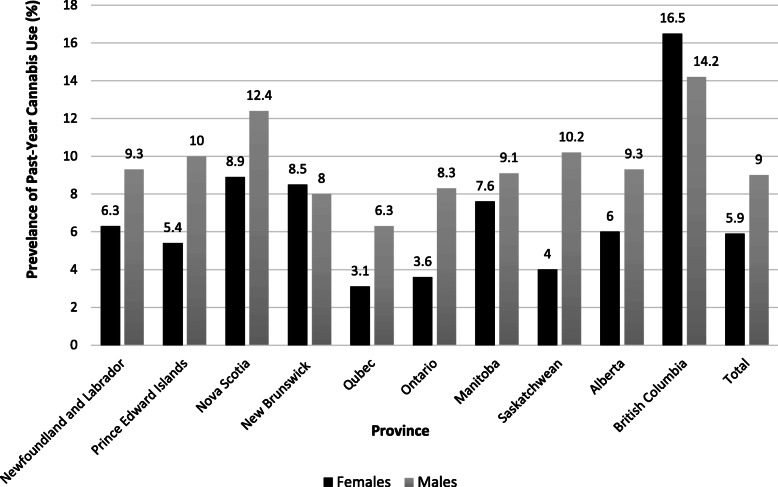
The prevalence of cannabis use among middle and older aged males and females over the past-year in the Canadian provinces, based on Canadian Tobacco, Alcohol and Drugs Survey, 2017

The prevalence of past-year male and female cannabis use, for all 10 provinces, are displayed in Fig. 1. When examining all the provinces in Canada, British Columbia was noted as having the highest prevalence of past-year cannabis use in both sexes (16.5% females vs. 14.2% males) followed by Nova Scotia (8.9% female vs. 12.4% male). Contrarily, the lowest prevalence of female and male past-year cannabis use was reported in Québec (3.1% vs. 6.3%) and Ontario (3.6% female vs. 8.3% male).

### Males

Results of the supplementary statistics are reported in Table 1. Over 27% of past-year cannabis users report using cannabis less than monthly, while 25.5% of users admit to daily or almost daily use. Overall 39.1% of male’s self-report using cannabis for medical purposes. Of these users, 63.5% report using cannabis for chronic pain, followed by 10.1% for insomnia, and 7.4% for anxiety/nerves. The primary way of accessing cannabis was through family members, shared with friends, or grown on their own. Additionally, 61.9% of males reported a failed attempt at reducing or stopping cannabis use over the past 3 months.

**Table 1:** Supplementary characteristics (weighted) of cannabis use among middle and older aged males and females who have consumed cannabis over the past-year, based on the 2017 Canadian Tobacco, Alcohol and Drugs Survey

	Middle and Older Aged Males % (N)	Middle and Older Aged Females % (N)
**Frequency of cannabis use (past 3 months)**		
Less than monthly	27.1 (205,294)	21.1 (114,796)
Monthly	7.7 (58,193)	14.1 (76,716
Weekly	19.8 (150,466)	15.5 (84,189)
Daily or almost daily	25.5 (193,575)	30.9 (167,851)
Not in the past 3 months	19.9 (7,976,914)	18.4 (8,806,401)
**Self-reported cannabis used for medical purposes**		
Yes	39.1 (301,447)	56.0 (307,751)
No	60.9 (469,543)	44.0 (241,833)
**Main self-reported medical conditions cannabis is used for**		
Chronic Pain (arthritis, back pain, migraines)	63.5 (191,382)	52.2 (160,644)
Depression	1.9 (5754)	2.1 (6509)
Multiple Sclerosis/Spinal Cord Injury	1.7 (5094)	8.6 (26,478)
Anxiety/Nerves	7.4 (22,191)	3.9 (12,126)
Insomnia	10.1 (30,565)	21.6 (66,536)
Other	15.4 (46,461)	11.5 (35,285)
**Where cannabis is obtained**		
Grow my own	11.2 (80,975)	4.0 (21,118)
Someone grows it for me (medical or recreational)	2.2 (15,992)	1.1 (5889)
Shared around a group of friends	11.4 (82,593)	3.4 (18,346)
From family members	39.6 (286,431)	49.3 (262,171)
From someone else I know	6.6 (47,592)	6.5 (34,474)
From a dealer (unlicensed)	8.5 (61,623)	5.2 (27,631)
From a licenced medical dealer	2.9 (21,128)	10.1 (53,880)
From a dispensary/compassion club	12.6 (91,271)	14.8 (78,975)
Online	0.5 (3508)	0.7 (3898)
Other	4.4 (31,481)	4.8 (25,505)
**How cannabis is consumed**		
Smoked a joint, bong, pipe or blunt		
Yes	89.0 (674,682)	74.3 (404,888)
No	11.0 (83,645)	25.7 (140,164)
Mixed with Tobacco (also known as spliff)		
Yes	19.5 (148,241)	13.9 (75,306)
No	80.5 (610,085)	86.1 (464,834)
Eaten in foods (ex. brownies, cake, cookies or candy)		
Yes	33.1 (250,761)	45.0 (245,161)
No	66.9 (507,566)	55.0 (299,891)
Drank in tea, cola, alcohol or other drinks		
Yes	6.0 (45,226)	3.1 (16,816)
No	94.0 (713,101)	96.9 (528,236)
Vaporized with a vaporiser, vape pen or e-cigarette		
Yes	22.4 (170,205)	28.8 (157,137)
No	77.6 (588,122)	71.2 (387,915)
Dabbed		
Yes	8.7 (66,045)	4.3 (23,188)
No	91.3 (690,708)	95.7 (521,863)
**Tried and failed to reduce/stop cannabis use (past 3 months)**		
Yes	61.9 (31,005)	11.6 (284)
No	38.1 (19,110)	88.4 (2156)
**Mean age at first use**		
	21.04	24.28

After adjusting for all covariates, statistically significant predictors for male past-year cannabis use are displayed in Table 2. Individuals without a partner were 2.13 times more likely to be past-year cannabis users, as compared to those with partners (OR: 2.13, 95% CI: 1.1–4.1). Additionally, those who smoked traditional tobacco cigarettes were 4.51 times more likely to be past-year cannabis users, compared to their counterparts (OR: 4.51, 95% CI: 2.5–8.2). Most notably, illicit drug users were 40.40 times more likely to be past-year cannabis users, compared to those who did not use illicit drugs (OR: 40.40, 95% CI: 10.0–162.6). There was no concern for multicollinearity as tested by bivariate correlations. The model fit for the middle and older adult male model was 0.23.

**Table 2:** Frequencies, unadjusted and adjusted odds ratios (ORs), along with corresponding 95% confidence intervals (95% CIs) and *p*-values of consuming cannabis in the past-year in middle and older aged *males*, aged 40 years and older, based on the Canadian Tobacco, Alcohol and Drugs Survey, 2017

	Unweighted N (%)	Weighted N (%)	% of Male Cannabis Use in the past-year	Male Cannabis Use in the past-year Unadjusted OR (95% CI)	*P* value	Male Cannabis Use in the past-year Adjusted OR (95% CI)	*P* value
**Sociodemographic Factors**							
**Age**							
40–49	560 (26.9)	2,354,362 (26.45)	9.61	2.55 (0.7–9.2)	0.15	1.50 (0.3–6.7)	0.54
50–59	787 (37.7)	2,635,059 (29.61)	12.29	**3.36 (1.0–11.1)**	**0.05**	2.40 (0.6–10.1)	0.23
60–69	452 (21.7)	2,125,741 (23.88)	8.21	2.14 (0.6–7.6)	0.24	1.50 (0.3–6.7)	0.60
70+	286 (13.7)	1,784,849 (20.05)	4.01	1		1	
**Province**^a^							
Western-BC	187 (9.0)	1,213,280 (13.63)	14.22	**2.04 (1.0–4.3)**	**0.05**	2.16 (0.9–5.0)	0.07
Western-Parries	470 (22.5)	1,508,518 (16.95)	9.44	1.28 (0.7–2.4)	0.45	1.15 (0.6–2.4)	0.70
Eastern-Atlantic	714 (34.2)	635,113.17 (7.14)	10.15	1.39 (0.8–2.4)	0.24	0.92 (0.4–1.9)	0.82
Central	714 (34.2)	5,543,098 (62.28)	7.51	1		1	
**Community Dwelling**							
Rural	626 (30.0)	2,065,634 (23.21)	11.85	1		1	
Urban	1459 (70.0)	6,834,376 (76.79)	8.12	1.07 (0.74–1.56)	0.17	0.56 (0.3–1.2)	0.14
**Marital Status**							
Partner	513 (24.7)	7,065,351 (79.83)	6.92	1		1	
No Partner	1561 (75.3)	1,784,707 (20.17)	17.26	**2.81 (1.6–4.8)**	**< 0.001**	**2.13 (1.1–4.1)**	**0.03**
**Indigenous Status**							
Yes	98 (4.8)	260,055 (3.02)	22.56	**3.10 (1.0–9.4)**	**0.05**	2.62 (0.8–8.7)	0.12
No	1941 (95/2)	8,340,211 (96.98)	8.60	1		1	
**Education**							
University and above	366 (20.1)	2,830,979 (33.11)	9.95	1		1	
Trade/College	693 (38.1)	2,605,917 (30.48)	8.01	0.78 (0.4–1.5)	0.48	0.53 (0.3–1.1)	0.08
Secondary	487 (26.8)	2,055,419 (24.04)	7.84	0.77 (0.4–1.6)	0.49	0.52 (0.2–1.2)	0.13
Less than Secondary	273 (15.0)	1,056,829 (12.36)	10.40	1.05 (0.5–2.5)	0.91	0.88 (0.3–2.6)	0.82
**Current Employment**							
Not Employed	755 (37.0)	3,662,317 (42.57)	7.88	1		1	
Employed	1285 (63.0)	4,940,932 (57.43)	9.82	1.27 (0.7–2.2)	0.39	1.34 (0.7–2.7)	0.41
**Health Factors**							
**Self-perceived Health**							
Very Good/Excellent	1214 (58.4)	5,074,587 (57.20)	7.95	1		1	
Good	632 (30.4)	2,881,546 (32.48)	11.02	1.43 (0.8–2.5)	0.20	1.04 (0.5–2.3)	0.93
Fair/Poor	233 (11.2)	915,816 (10.32)	8.46	1.07 (0.5–2.2)	0.86	0.76 (0.2–2.5)	0.65
**Self-perceived Mental Health**							
Very Good/Excellent	1480 (71.2)	6,351,499 (71.64)	7.14	1		1	
Good	508 (24.4)	2,096,547 (23.65)	12.71	**1.89 (1.1–3.3)**	**0.03**	1.65 (0.7–4.0)	0.26
Fair/Poor	92 (4.4)	418,163 (4.72)	16.92	**2.65 (1.0–6.9)**	**0.05**	3.44 (0.8–14.0)	0.09
**Substance Use Variables**							
**Drink Alcohol**							
No	418 (20.4)	1,826,839 (21.01)	5.49	1		1	
Yes	1636 (79.6)	6,866,838 (78.99)	9.92	1.90 (0.8–4.2)	0.12	2.09 (0.8–5.7)	0.15
**Smoke Cigarette**							
No	1749 (83.9)	7,461,424 (83.84)	5.70	1		1	
Yes	336 (16.1)	1,438,585 (16.16)	26.80	**6.05 (3.5–10.5)**	**< 0.001**	**4.51 (2.5–8.2)**	**< 0.001**
**Other Tobacco Products**^b^							
No	1952 (94.0)	8,404,912 (94.75)	8.19	1		1	
Yes	125 (6.0)	465,582 (5.25)	23.77	**3.50 (1.6–7.5)**	**< 0.001**	1.26 (0.5–3.2)	0.63
**Illicit Drug Use**^c^							
No	1974 (98.5)	8,341,756 (98.63)	8.06	1		1	
Yes	31 (1.5)	115,470 (1.37)	82.79	**54.87 (13.7–219.1)**	**< 0.001**	**40.40 (10.0–162.6)**	**< 0.001**

^a^Western-BC category includes: British Columbia; Western Prairies category includes: Manitoba, Saskatchewan, & Alberta; Eastern Atlantic category includes: Newfoundland & Labrador, Nova Scotia, Prince Edward Island & New Brunswick; and Central category includes Quebec & Ontario

^b^Includes: Cigarillo, Cigar, Tobacco Water-pipe and Smokeless tobacco

^c^Includes: cocaine, speed/meth, ecstasy, hallucinogens, salvia, heroin, inhalants, abuse of pain relievers, stimulants and sedatives to get high in the past-year

### Females

Of past-year cannabis users, over 30% report daily or almost daily use (Table 1). Among females, 56% self-reported using cannabis for medical reasons. Of self-reported medical cannabis users, 52.2% reported its use for chronic pain, 21.6% for insomnia, and 8.6% for pain associated with multiple sclerosis/spinal cord injury. The primary method of accessing cannabis was through family members, from a dispensary, or a licenced medical dealer.

After adjusting for all covariates (Table 3), significant sociodemographic factors of past-year cannabis use among females included residing in British Columbia (OR: 5.50, 95% CI: 2.4–12.8), or Eastern-Atlantic provinces (OR: 2.63, 95% CI: 1.4–4.9) as compared to the central provinces. Residing in an urban community also showed strong statistical association with past-year cannabis use (OR: 2.21, 95% CI: 1.24–3.91) when compared to living in a rural community. Additionally, among health factors**,** having fair or poor mental health was associated with over a four-fold greater risk of using cannabis in the past-year (OR: 4.29, 95% CI: 1.1–16.3) as compared to having excellent or very good mental health. When looking at substance use variables, current cigarette smoking was identified as a significant predictor for being a past-year cannabis user as compared to those who did not smoke cigarettes (OR: 2.90, 95% CI: 1.5–5.6). Moreover, females who reported using other tobacco products were 7.55 times more likely to be past-year cannabis users, compared to those who did not use other tobacco products (OR: 7.55, 95% CI: 2.3–25.0). Finally, illicit drug users were also at a significantly greater odds be past-year cannabis users, compared to non-drug users (OR: 6.99, 95% CI: 1.2–41.8). There was no concern for multicollinearity as tested by bivariate correlations. The model fit for the middle and older aged female model was 0.30.

**Table 3:** Frequencies, unadjusted and adjusted odds ratios (ORs), along with corresponding 95% confidence intervals (95% CIs) and p-values of consuming cannabis in the past-year in middle and older aged *females*, aged 40 years and older, based on the Canadian Tobacco, Alcohol and Drugs Survey, 2017

	Unweighted N (%)	Weighted N (%)	% Female Cannabis Use in the past-year	Female Cannabis Use in the past-year Unadjusted OR (95% CI)	*P* value	Female Cannabis Use in the past-year Adjusted OR (95% CI)	*P*- Value
**Sociodemographic Factors**							
**Age**							
40–49	839 (31.0)	2,372,501 (25.23)	7.57	2.58 (0.8–8.2)	0.11	2.51 (0.6–10.3)	0.20
50–59	993 (36.7)	2,643,036 (28.11)	8.35	**4.37 (1.5–13.1)**	**0.01**	3.62 (0.9–15.2)	0.08
60–69	463 (17.1)	2,204,961 (23.45)	5.11	**3.92 (1.3–11.9)**	**0.02**	2.11 (0.5–8.7)	0.30
70+	409 (15.1)	2,182,059 (23.21)	2.04	1		1	
**Province**^a^							
Western-BC	257 (9.5)	1,284,160 (13.66)	16.50	**5.59 (2.5–12.7)**	**< 0.001**	**5.50 (2.4–12.8)**	**< 0.001**
Western-Parries	614 (22.7)	1,520,428 (16.17)	5.94	**1.78 (1.0–3.3)**	**0.06**	1.32 (0.7–2.6)	0.41
Eastern-Atlantic	937 (34.7)	686,535 (7.30)	7.95	**2.44 (1.4–4.2)**	**< 0.001**	**2.63 (1.4–4.9)**	**< 0.001**
Central	896 (33.1)	5,911,434 (62.87)	3.41	1		1	
**Community Dwelling**							
Rural	741 (27.4)	2,148,670 (22.85)	3.08	1		1	
Urban	1963 (72.6)	7,253,887 (77.15)	6.78	**2.29 (1.40–3.74)**	**< 0.001**	**2.21 (1.24–3.91)**	**0.01**
**Marital Status**							
Partner	1004 (37.4)	6,247,035 (66.82)	5.56	1		1	
No Partner	1677 (62.6)	3,101,371 (33.18)	6.75	1.23 (0.7–2.2)	0.48	1.37 (0.7–2.6)	0.32
**Indigenous Status**							
Yes	113 (4.3)	348,095 (3.80)	9.28	1.64 (0.5–5.1)	0.40	1.95 (0.6–5.9)	0.24
No	2538 (95.7)	8,802,025 (96.20)	5.88	1		1	
**Education**							
University degree and above	534 (22.1)	2,427,296 (26.46)	4.46	1		1	
Trade/College	1026 (42.4)	3,610,170 (39.36)	6.79	1.56 (0.9–2.8)	0.14	1.47 (0.7–2.9)	0.27
Secondary	631 (26.1)	2,211,016 (24.10)	7.87	1.83 (0.8–4.2)	0.15	1.52 (0.6–4.0)	0.40
Less than Secondary	226 (9.4)	924,819 (10.08)	2.43	0.53 (0.1–2.3)	0.39	0.66 (0.1–4.4)	0.67
**Current Employment**							
Not Employed	1192 (44.8)	5,222,818 (56.43)	4.99	1		1	
Employed	1470 (55.2)	4,031,823 (43.57)	7.17	1.47 (0.8–2.7)	0.22	1.29 (0.6–2.8)	0.54
**Health Factors**							
**Self-perceived Health**							
Very Good/Excellent	1674 (62.0)	5,755,823 (61.28)	5.49	1		1	
Good	722 (26.7)	2,505,452 (26.67)	5.76	1.05 (0.6–2.0)	0.87	0.95 (0.5–1.8)	0.88
Fair/Poor	306 (11.3)	1,132,105 (12.05)	8.68	1.63 (0.8–3.4)	0.18	1.17 (0.4–3.5)	0.78
**Self-perceived Mental Health**							
Very Good/Excellent	1927 (71.5)	6,824,680 (72.85)	4.56	1		1	
Good	622 (23.0)	2,048,872 (21.87)	7.06	1.59 (0.9–2.9)	0.14	1.35 (0.7–2.6)	0.37
Fair/Poor	147 (5.5)	495,105 (5.28)	21.31	**5.67 (1.6–20.3)**	**0.01**	**4.29 (1.1–16.3)**	**0.03**
**Substance Use Variables**							
**Drink Alcohol**							
No	687 (25.8)	2,276,016 (24.59)	3.88	1		1	
Yes	1975 (74.2)	6,978,702 (75.41)	6.57	1.74 (0.8–3.7)	0.15	1.72 (0.7–4.0)	0.21
**Smoke Cigarette**							
No	2322 (85.9)	8,179,106 (85.46)	3.88	1		1	
Yes	382 (14.1)	1,223,451 (13.01)	19.87	**6.15 (3.1–12.1)**	**< 0.001**	**2.90 (1.5–5.6)**	**< 0.001**
**Other Tobacco Products**^b^							
No	2631 (97.5)	9,240,658 (98.38)	5.43	1		1	
Yes	67 (2.5)	152,494 (1.62)	36.34	**9.94 (3.5–27.9)**	**< 0.001**	**7.55 (2.3–25.0)**	**< 0.001**
**Illicit Drug Use**^c^							
No	2599 (98.8)	9,052,725 (98.89)	5.67	1		1	
Yes	31 (1.2)	80,090 (0.88)	44.69	**13.45 (2.0–92.1)**	**< 0.001**	**6.99 (1.2–41.8)**	**0.03**

^a^Western-BC category includes: British Columbia; Western Prairies category includes: Manitoba, Saskatchewan, & Alberta; Eastern Atlantic category includes: Newfoundland & Labrador, Nova Scotia, Prince Edward Island & New Brunswick; and Central category includes Quebec & Ontario

^b^Includes: Cigarillo, Cigar, Tobacco Water-pipe and Smokeless tobacco

^c^Includes: cocaine, speed/meth, ecstasy, hallucinogens, salvia, heroin, inhalants, abuse of pain relievers, stimulants and sedatives to get high in the past-year

## Discussion

The aim of this study was to assess the prevalence, characteristics, and patterns of pre-legalization cannabis use among middle and older aged Canadians, independently for males and females. In 2017, a year before Canadian cannabis legalization, the overall prevalence of past-year cannabis use in middle and older aged adults was 7.4%, which was higher than an earlier analysis conducted in 2012 [16]. In the present study, 9.0% males and 5.9% of females reported cannabis use in the past year. When examining the older male subgroup closer, determinants of use include having no partner, being a current cigarette smoker, and being an illicit drug user. Characteristics for older females, include residing in British Columbia or the Eastern-Atlantic provinces, residing in an urban community, suffering from poor mental health, cigarette smoking, using other tobacco products, and illicit drug use. This study clearly illustrates the sex-specific differences in cannabis use among older Canadians, providing baseline data for future studies investigating substance use and disorders among this age cohort and between sexes.

Middle and older aged males reported an increased overall prevalence of past-year cannabis use in 2017. While increased prevalence of cannabis among males was also recently reported in the United States by Hasin et al. [33] the reason for this finding might be multi-factorial. Although specific reasons remain debated, it is possible that cultural and behavioural acceptance of learned smoking habits in the male subgroups could be a contributing factor of increased cannabis use [34]. Additionally, it is interesting that our results note 62% of males who tried to quit or reduce their cannabis intake over the past 3 months, failed to do so (Table 1). These results are in agreeance with other investigations, which note that males have an increased prevalence of cannabis addiction and cannabis use disorders, making it difficult to quit overall cannabis intake [33, 35]. However, a study conducted by Herrmann (2015) highlighted that females were more likely to show more individual withdrawal symptoms, a higher severity, and more discomfort during the cannabis withdrawal process, when compared to males [36]. With increasing rates of cannabis use among females, it would be interesting to examine whether these trends hold true to the older Canadian sub-sample.

Supplementary characteristics note a high percentage (56%) of self-reported female medical cannabis users. This is in line with research in rat models that have found female rats to be more sensitive to Tetrahydrocannabinol’s (THC) pain blocking effects as compared to their male counterparts [37]. Although previous studies have identified that medical marijuana use is more commonly seen in men [38–40], the trend of heightened mental health disorders among women may be the cause for the increased uptake of medical marijuana use in this population [41]. Additionally, it is important to note the rising popularity of vaporizers, such as e-cigarettes, as a delivery tool among this sample (Table 1). The increased prevalence of e-cigarette use has been noted across all age groups and should be monitored carefully given the rise in severe respiratory illnesses in relation to the concomitant use of e-cigarettes and cannabis products [28, 42–44].

Among sociodemographic factors, having no partner was a determinant for past-year cannabis use among males. While many studies have reported marital and relationship status to be a general predictor of substance use in all sexes [33, 45–47], it is important to note that no other studies have identified such an association to be specific to males. Many factors including the lack of social support, increased leisurely time allowing for more experimental behaviors, and lack of familial commitments, could be contributing to this association [46, 47]. Having said that, more exploration on the role that marital and romantic relationships in regulating drug usage is necessary.

In females, sociodemographic factors such as residing in urban communities was associated with past-year cannabis use. Hasin et al. (2018) has confirmed the notion of increased cannabis availability and subsequent usage in urban regions [33]. Additionally, a sex-based difference was noted among Slovakian females, that showed riskier substance use in females who reside in urban areas [48]. This study also highlighted that females residing in either British Columbia or the Eastern-Atlantic Provinces are more likely to be past-year cannabis users, compared to the other Canadian provinces. This finding is supported by a previous report by Statistics Canada, which found that British Columbians and Nova Scotians (one of four Eastern-Atlantic Provinces) had significantly higher prevalence of past-year marijuana use compared to other Canadian provinces [16]. Although Canada is one of the largest cannabis cultivators in the world, large quantities of cannabis have been known to be smuggled into Canada through Canadian seas and ports [49–51]. Therefore, the close proximity of British Columbia and Nova Scotia to international shipping ports may make them prime location for illegally shipped contraband.

In regard to health factors, our findings indicate that females who experienced poorer self-reported mental health were more likely to be cannabis users as compared to those who reported very good or excellent mental health. An American study noted that mental well-being among cannabis users was significantly poorer than their non-using counterparts, especially among female users [52]. Although cannabis has been shown to have modest health benefits, long term use has been associated with cognitive impairment [53–55]. The association between using cannabis and adverse mental health outcomes, such as increased depressive symptomology, worsening anxiety, and panic disorders, have been reported previously [41, 56]. Given that cannabis is often portrayed as a tool to improve mental health, adult cannabis users were likely to over-acknowledge its potential benefits [57, 58]. Therefore, these findings can also be indicative of individuals using cannabis products to manage declining mental health, as a form of self-therapy or prescribed rehabilitation [59]. Given the ambiguity surrounding the long-term effects of cannabis, it is important that public health professionals ensure that using cannabis does not exacerbate existing mental health problems or replace healthier mental health treatments.

Finally, among substance use variables, this study showed increased cannabis use among both male and female illicit drug users. In particular, a study by Han et al. (2017) highlighted the alarming rates of illicit drug use, in association with cannabis use in older populations [60]. While cannabis being a gateway drug into illicit drug use remains highly debated [61–66], there is a general agreement among researchers to monitor the transition of marijuana to more lethal drugs [65–67]. Given that the simultaneous use of multiple substances can make older adults susceptible to declining executive functions [68, 69], it is essential that measures to combat multiple substance use disorders are implemented to prevent additional burden of disease among this population.

Although novel and imperative to the body of substance use literature, this study is subjected to a few limitations. The CTADS relies on self-reported data and therefore is subjected to recall bias. This survey is also unable to capture some important variables including ethnicity and income, which may have influenced cannabis use behaviors in this sample. As is the case for all cross-sectional analyses, causational interpretation of these findings cannot be determined. The CTADS also neglects to include those living on the three Canadian territories and institutionalized populations, including those that are incarcerated, institutionalized, or active in the military. Some of these populations are identified in the literature to be active and substantial users of cannabis, and therefore their exclusion could have altered overall prevalence rates. Regardless of these limitations, this study allows for a large sample size and provides key insights on sex specific trends associated with cannabis use prior to legalization in Canada.

## Conclusion

To our knowledge, this is the first nationally representative Canadian study to outline the prevalence, characteristics, and patterns of cannabis use in middle and older aged adults. In specific, predictors of past-year cannabis use for older and middle-aged males included having no partner, being a current cigarette smoker and illicit drug user. Predictors for females included residing in British Columbia, in the Eastern-Atlantic provinces and in an urban community, having poor mental health, smoking cigarettes, using tobacco products, and illicit drugs. This study highlights the importance of including middle and older aged adults in the discussion around cannabis, in order to create awareness around its use. Results from this study can help identify longitudinal trends of using cannabis in regard to the pre and post legalization era of cannabis in Canada. With the current landscape, it is important to further investigate the benefits of regular cannabis use among older Canadians, as well as identify potentials for harms in situations of misuse, overuse, and dependency. Additionally, our results suggest more research is needed around the female mental health and cannabis use, specifically the use of marijuana as a replacement for other beneficial mental health treatments and the potential trigger of worsening symptomology. Furthermore, as the population continues to age, it is important to examine substance use in this group distinctly and implement initiatives to offset any associated comorbidities.

## Data Availability

The data collected by Statistics Canada as part of the CTADS 2017 can be accessed through a formal application submitted to Statistics Canada via their website at http://www23.scensus-recensement/imdb/p2SV.pl?Function=getSurvey&SDDS=4440.

## Abbreviations

CUD: Cannabis Use Disorder
CTADS: Canadian Tobacco, Alcohol and Drugs Survey
OR: Odds Ratio
CI: Confidence Interval
